# Effects of different alkylating agents on photoreceptor degeneration and proliferative response of Müller glia

**DOI:** 10.1038/s41598-023-50485-7

**Published:** 2024-01-02

**Authors:** Kaori Nomura-Komoike, Reiko Nishino, Hiroki Fujieda

**Affiliations:** 1 Department of Anatomy and Neurobiology, School of Medicine, Tokyo Women’s Medical University, 8-1 Kawada-cho, Shinjuku-ku, Tokyo, 162-8666 Japan; 2 Department of Ophthalmology, School of Medicine, Tokyo Women’s Medical University, Tokyo, Japan

**Keywords:** Neuroscience, Neurology

## Abstract

Animal models for retinal degeneration are essential for elucidating its pathogenesis and developing new therapeutic strategies in humans. *N*-methyl-*N*-nitrosourea (MNU) has been extensively used to construct a photoreceptor-specific degeneration model, which has served to unveil the molecular process of photoreceptor degeneration as well as the mechanisms regulating the protective responses of remaining cells. Methyl methanesulphonate (MMS), also known to cause photoreceptor degeneration, is considered a good alternative to MNU due to its higher usability; however, detailed pathophysiological processes after MMS treatment remain uncharacterized. Here, we analyzed the time course of photoreceptor degeneration, Müller glial proliferation, and expression of secretory factors after MNU and MMS treatments in rats. While the timing of rod degeneration was similar between the treatments, we unexpectedly found that cones survived slightly longer after MMS treatment. Müller glia reentered the cell cycle at a similar timing after the two treatments; however, the G1/S transition occurred earlier after MMS treatment. Moreover, growth factors such as FGF2 and LIF were more highly upregulated in the MMS model. These data suggest that comparative analyses of the two injury models may be beneficial for understanding the complex regulatory mechanisms underlying the proliferative response of Müller glia.

## Introduction

Retinal degenerative diseases such as retinitis pigmentosa and age-related macular degeneration are the leading causes of blindness. The pathogenic mechanisms of these diseases are not fully understood and no effective treatments are currently available. Animal models are essential for our understanding of the pathogenesis of retinal diseases and the development of new therapeutic strategies in humans. A number of animal models for studying retinal degeneration are available including those induced by physical or chemical treatments, natural mutants, or genetically engineered animals. Among them, retinal injury induced by DNA alkylating agents, such as *N*-methyl-*N*-nitrosourea (MNU), has been widely used as a model of retinitis pigmentosa^1,2^. A single systemic administration of MNU induces highly reproducible photoreceptor-specific degeneration in a variety of animal species at a desired time^1^. This chemically induced retinal injury model has proven useful for understanding the process of photoreceptor degeneration as well as the injury-induced responses of other retinal cells such as Müller glia^1,3–5^.

Müller glia, the principal glial cell type in the retina, play important roles in maintaining retinal homeostasis under both physiological and pathological conditions^6^. In response to retinal injury, Müller glia in zebrafish proliferate, dedifferentiate into neuronal progenitors, and regenerate lost neurons while in mammals, Müller glia hardly proliferate and undergo reactive gliosis instead of neuronal regeneration^7^. We have reported the mechanisms regulating the injury-induced responses of mammalian Müller glia using the rat model of MNU-induced photoreceptor injury^3,4^. Rat Müller glia are capable of proliferation after injury, but many of the progeny of Müller glia divisions die likely due to the DNA damage response^3^. We also showed that phosphatidylserine and Rac1, key regulators of the phagocytic pathway, control the phagocytic activity, proliferation, migration, and reactive gliosis of rat Müller glia after MNU-induced injury^4^.

Methyl methanesulfonate (MMS), another DNA alkylating agent, has been shown to induce photoreceptor-specific degeneration similar to the MNU-induced model^8^ . Although the mechanisms underlying MMS-induced photoreceptor death have been well investigated^8–10^, detailed pathophysiological processes of MMS-induced retinal injury including the timing of photoreceptor cell death and Müller glia proliferation remain unexplored. We thus sought to characterize the MMS-induced photoreceptor injury in comparison with the MNU model to gain insights into the mechanisms regulating injury-induced Müller glia proliferation. The timing of G1 phase entry of Müller glia was similar between the MNU and MMS models, but we unexpectedly found that the G1/S transition was more accelerated in the MMS model. The timing of rod death was similar between the models while cone death was slightly delayed in the MMS model. The levels of growth factor expression were also different between the MNU and MMS models. Our data reveal unexpected differences in the pathophysiological processes of the MNU and MMS models and provide evidence for mechanistic links between photoreceptor death, activation of secretory factors and cell cycle progression of Müller glia.

## Results

### Photoreceptor degeneration is induced by MNU and MMS in a dose-dependent manner

**Figure 1:**
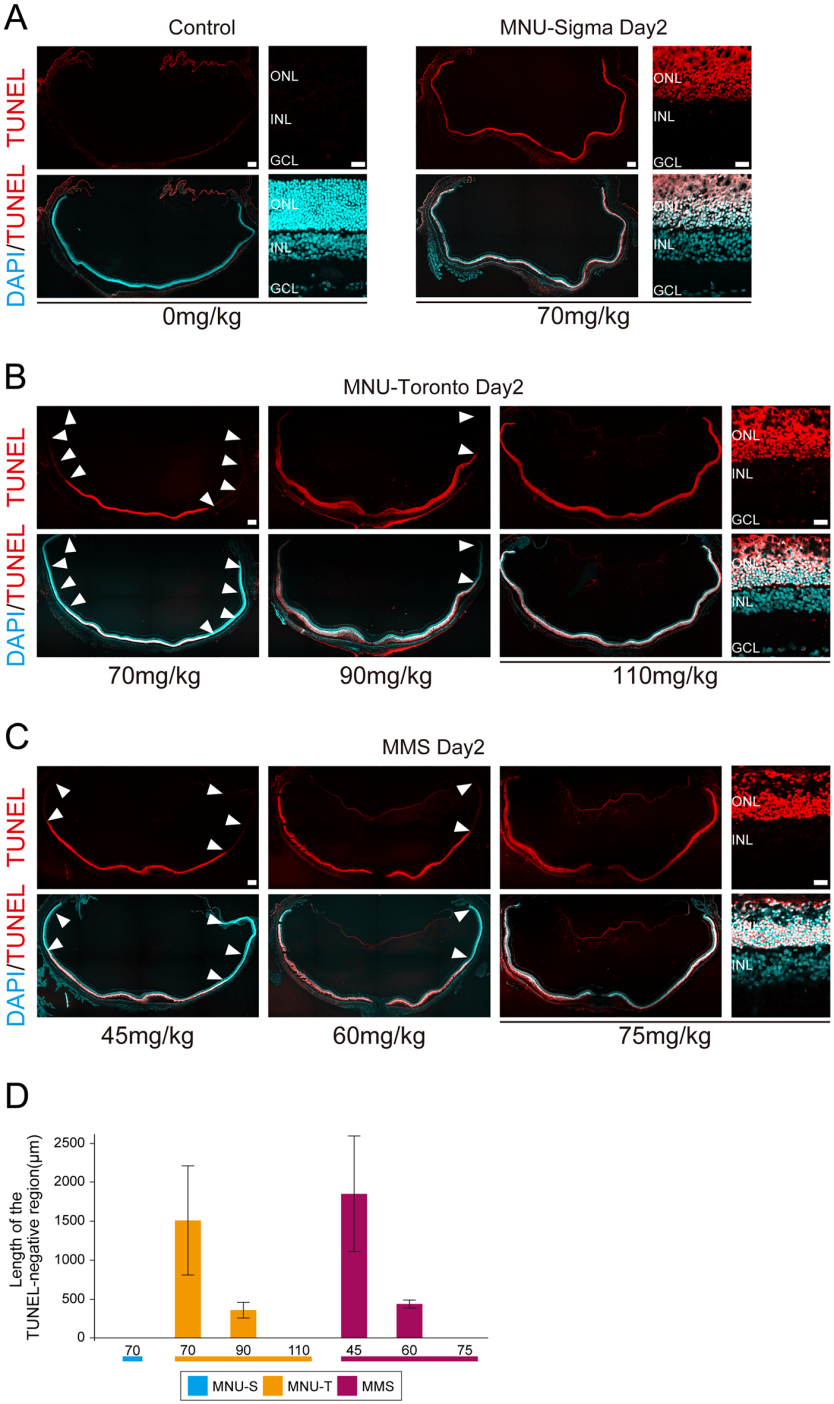
Dose-dependent photoreceptor degeneration after treatment with alkylating agents. TUNEL assay (red) in the non-treated control and the retinas treated with MNU-S (**A**), MNU-T (**B**), and MMS (**C**) at the given doses. Cross sections of the entire retinas and the higher magnifications of the central regions are shown. White arrowheads indicate TUNEL-negative areas, the length of which was quantitated and shown as graphs (**D**). Each bar represents the mean ± standard deviation (SD, n = 3). Cell nuclei are counterstained with DAPI (cyan). *ONL* outer nuclear layer, *INL* inner nuclear layer, *GCL* ganglion cell layer. Scale bars are 200 µm and 20 µm for lower and higher magnifications, respectively.

We first conducted the TUNEL assay to compare the effects of two alkylating agents, MNU and MMS, to induce photoreceptor degeneration in rats. MNU was obtained from two commercial sources, Sigma-Aldrich (St. Louis, MO, USA) and Toronto Research Chemical (Toronto, ON, Canada), and hereafter referred to as MNU-S and MNU-T, respectively. MNU-S at the dose of 70 mg/kg BW induced photoreceptor degeneration in the whole retina from the central region to the periphery by day 2 after treatment (Fig. 1A), as reported previously^3^. We expected that the same dose of MNU-T would show an equivalent effect. However, 70 mg/kg BW MNU-T caused TUNEL-positive photoreceptor death only in the central retina by day 2 (Fig. 1B). Higher doses induced expansion of the TUNEL-positive area and 110 mg/kg BW of MNU-T generated the same extent of photoreceptor degeneration as induced by 70 mg/kg BW MNU-S. We tested three doses of MMS including 75 mg/kg BW, which has been used to induce photoreceptor degeneration in the mouse retina^8^. The outer nuclear layer (ONL) of the central retina was TUNEL-positive at day 2 regardless of dose, but only 75 mg/kg BW of MMS induced photoreceptor degeneration in the whole retina including the periphery (Fig. 1C). Quantification of the length of the TUNEL-negative regions of the peripheral retinas verified the results (Fig. 1D). We thus concluded that MNU-S (70 mg/kg BW), MNU-T (110 mg/kg BW), and MMS (75 mg/kg BW) induced the same extent of photoreceptor degeneration. These doses were used in subsequent experiments.

### G1/S transition of Müller glia occurs earlier in the MMS-treated retinas than MNU-treated retinas

**Figure 2:**
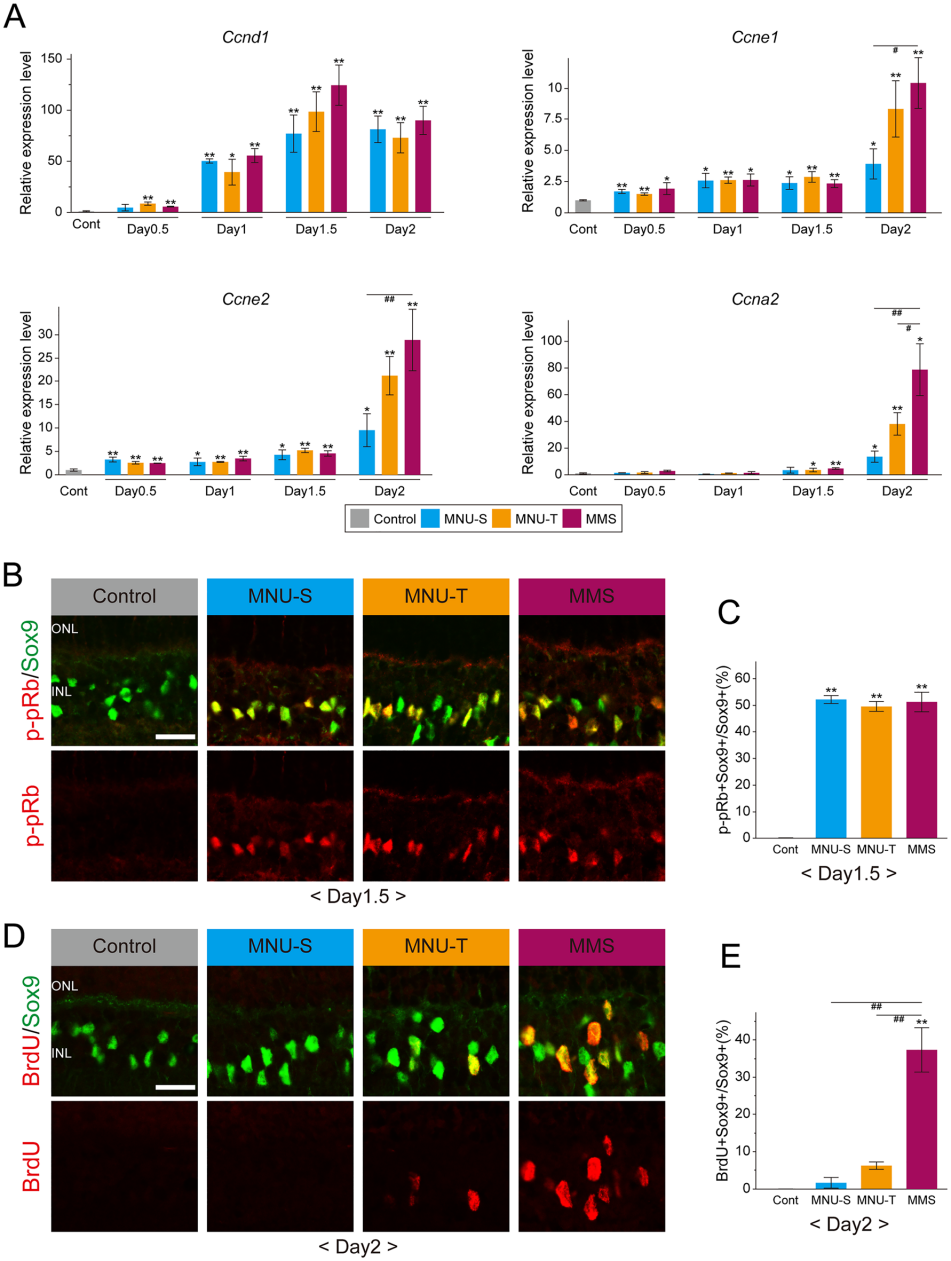
Cell cycle reentry and G1/S transition of Müller glia after MNU- and MMS-induced retinal injury. (**A**) qRT-PCR analyses of cell cycle-related genes (*Ccnd1*, *Ccne1*, *Ccne2*, and *Ccna2*) after MNU and MMS treatments. Each bar represents the mean ± standard deviation (SD, n = 3) and the values expressed relative to the controls (Cont) after normalization to *Gapdh* levels. (**B**) Double immunofluorescence for Sox9 (green) and p-pRb (red) at day 1.5 after treatments with the alkylating agents. (**C**) The proportions of p-pRb-positive Müller glia. (**D**) Double immunofluorescence for Sox9 (green) and BrdU (red) at day 2 after treatments with the alkylating agents. (**E**) The proportions of BrdU-positive Müller glia. Each bar of the graphs represents the mean ± SD (n = 3). *p < 0.05, **p < 0.01 (comparisons with control). ^#^*p* < 0.05, ^##^*p* < 0.01 (comparisons between treatments at the same stage). *ONL* outer nuclear layer, *INL* inner nuclear layer. Scale bar = 20µm.

We previously reported that photoreceptor injury induces rat Müller glia to reenter G1 phase of the cell cycle by day 2, followed by S phase entry by day 2.5, after MNU-S administration^3^. We thus tested whether MNU-T and MMS treatments trigger cell cycle reentry of Müller glia with a timing similar to MNU-S treatment. We first monitored expression of cell cycle-related genes including cyclin D1 (*Ccnd1*), the regulator of early G1 phase, cyclin E (*Ccne1* and *Ccne2*) regulating G1/S transition, and cyclin A2 (*Ccna2*) promoting S phase progression^11^, by quantitative RT-PCR (qRT-PCR). The *Ccnd1* expression significantly increased compared to controls after all treatments with peak levels at day 1.5 and did not vary significantly between treatments at any stages examined (Fig. 2A). The increase in the *Ccne1* and *Ccne2* levels were rather modest after all treatments and there were no significant differences between treatments until day 1.5 (Fig. 2A). Notably, however, the levels of *Ccne1* and *Ccne2* drastically increased by day 2 after MMS treatment, resulting in the significantly higher cyclin E levels in this model compared to the MNU-S model at day 2 (Fig. 2A). Similar to the cyclin E genes, the *Ccna2* levels were dramatically upregulated at day 2 after MNU-T and MMS treatments, when the *Ccna2* levels were significantly higher after MMS treatment compared to those after MNU treatments (Fig. 2A). We also tested whether the proliferating cell population in the injured retinas consisted exclusively of Müller glia and confirmed that most, if not all, phosphorylated retinoblastoma protein (p-pRb)-positive cells were Müller glia, but not astrocytes or microglia, at least for the experimental period examined (Supplemental Fig. 1A–C). We thus concluded that the expression patterns of the cyclin genes would reflect the cell cycle progression of Müller glia after injury.

We next analyzed the timing of cell cycle reentry of Müller glia by immunofluorescence for p-pRb (G1 phase entry) and bromodeoxyuridine (BrdU) incorporation assay (S phase entry). Consistent with the robust expression of *Ccnd1* at day1.5, approximately half of the Müller glia were positive for p-pRb at day 1.5 after all treatments (Fig. 2B, C). As expected, no p-pRb labeling was observed in controls (Fig. 2B, C) and at day 1 (Supplemental Fig. 1D). There were no significant differences in the proportion of p-pRb-positive Müller glia between treatments at day 1.5 (Fig. 2C). This indicates that a substantial number of Müller glia reenter the G1 phase by day 1.5 after all treatments. On the other hand, similarly to the expression of *Ccna2*, BrdU-positive Müller glia were not detected until day 2 (see Supplementary Fig. 1E showing the absence of BrdU signals at day 1.5 after all treatments), when approximately 40% of Müller glia were BrdU-positive in the MMS-treated retinas while few Müller glia were BrdU-positive after MNU-S and MNU-T treatments (Fig. 2D, E). These results suggest that G1/S transition of Müller glia occurs earlier in the MMS-treated retinas compared to the MNU-treated retinas, consistent with the temporal pattern of cyclin E and A upregulation (Fig. 2A).

### Cone photoreceptors survive longer in the MMS-treated retinas than MNU-treated retinas

**Figure 3:**
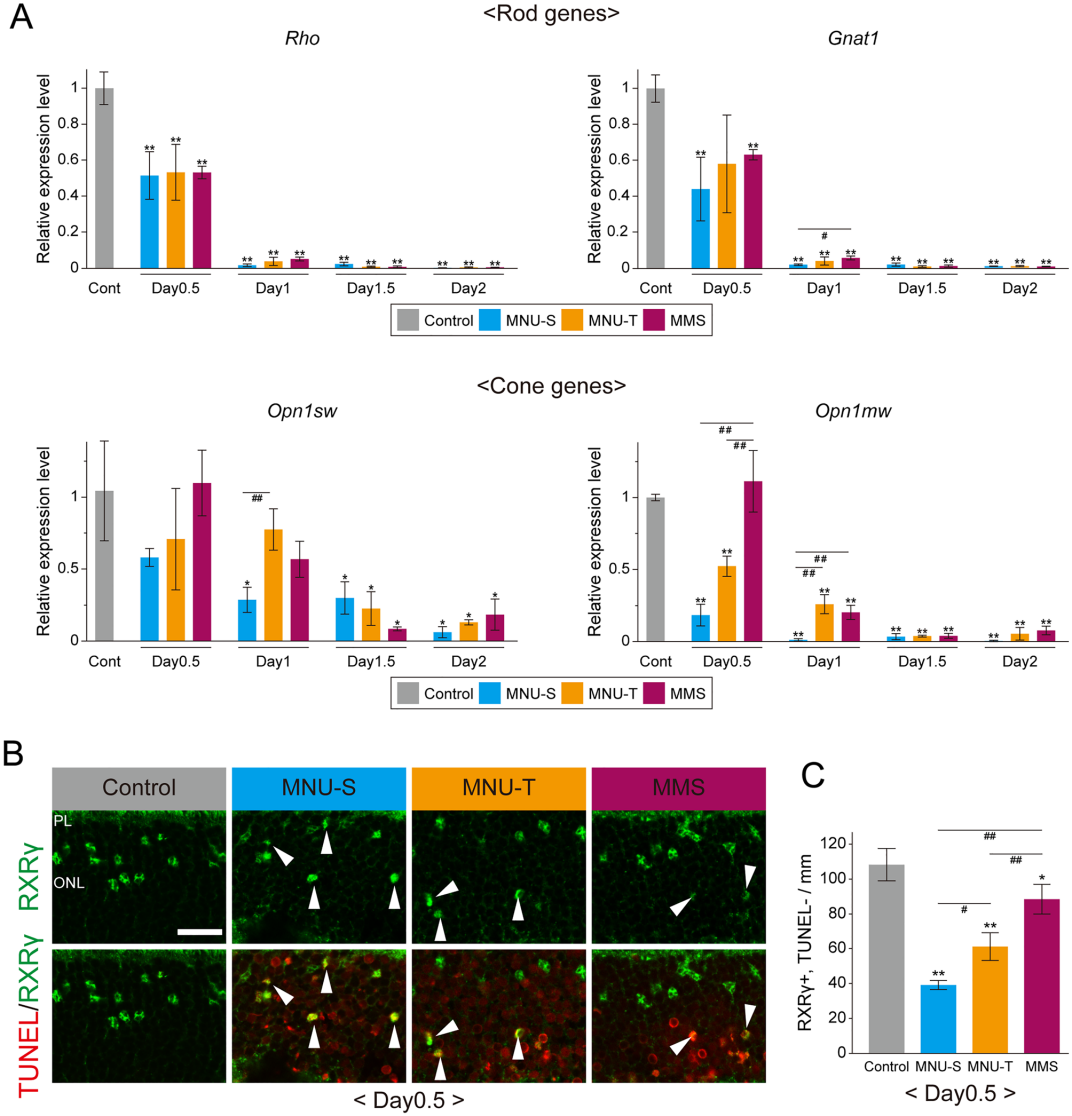
The time course of photoreceptor degeneration after treatment with alkylating agents. (**A**) qRT-PCR analyses of rod genes (*Rho* and *Gnat1*) and cone genes (*Opn1sw* and *Opn1mw*) after MNU and MMS treatments. (**B**) Immunofluorescence for RXRγ (green) in combination with TUNEL (red) at day 0.5 after treatment with alkylating agents. White arrowheads indicate double positive cells. *PL* photoreceptor layer, *ONL* outer nuclear layer. Scale bar = 20µm. (**C**) Quantification of surviving cone photoreceptors (RXRγ+, TUNEL−) at day 0.5 after MNU and MMS treatments. Each bar of the graphs represents the mean ± SD (n = 3). *p < 0.05, **p < 0.01 (comparisons with control). ^#^*p* < 0.05, ^##^*p* < 0.01 (comparisons between treatments at the same stage).

Earlier occurrence of G1/S transition of Müller glia after MMS treatment suggests that photoreceptor degeneration may occur earlier in the MMS-treated retinas. We thus examined in more detail the timing of photoreceptor degeneration by analyzing the expression of rod and cone-specific genes up to day 2. qRT-PCR data showed that the expression of two rod-specific genes, *Rho* and *Gnat1*, were significantly decreased to approximately half of control by day 0.5 and virtually lost by day 1 after all treatments (Fig. 3A). Contrary to our expectation, there were no significant differences in the levels of *Rho* and *Gnat1* expression between treatments at all stages examined, except a small difference in the *Gnat1* levels between MNU-S and MMS at day 1 (Fig. 3A). The temporal expression patterns of cone genes, *Opn1sw* and *Opn1mw*, were even more unexpected. At day 0.5, the expression of *Opn1sw* showed a trend to decrease after MNU-S treatment (*p* = 0.10) while no difference from control was observed after MNU-T and MMS treatment (Fig. 3A). The *Opn1mw* levels were significantly reduced by day 0.5 after MNU-S and MNU-T treatments (*p* < 0.01) but remained the control levels after MMS treatment (Fig. 3A). The *Opn1mw* levels were significantly higher in the MMS-treated retinas compared to the MNU-treated retinas at day 0.5 (Fig. 3A). The differences in cone gene expression across treatments were no more significant by day 1.5 (Fig. 3A). To confirm the expression pattern of cone genes, we further examined another cone-specific gene, *Gnat2*, by real-time PCR. Similar to the levels of *Opn1mw*, the *Gnat2* levels at day 0.5 after MMS treatment were kept at the control levels and significantly higher than those after MNU-S treatments (Supplemental Fig. 2A). These results indicate that rod degeneration occurs with a similar timing after all treatments while cone degeneration is slightly delayed after MMS treatment.

To confirm the qRT-PCR data, we performed immunofluorescence for retinoid X receptor γ (RXRγ), a cone photoreceptor marker, in combination with TUNEL. The density of RXRγ-positive, TUNEL-negative cells was calculated to evaluate the survival of cones. The density of surviving cones was lowest after MNU-S treatment and highest after MMS treatment at day 0.5 and day 1 (Fig. 3B, C, Supplementary Fig. 2B, C). The immunoreactivity of RXRγ was undetectable after day 1.5 (Supplemental Fig. 2D). Together, these results suggest that cones survive slightly longer after MMS treatment than MNU treatment.

### The expression levels of cytokines and growth factors were altered in the MNU- and MMS-treated retinas

**Figure 4:**
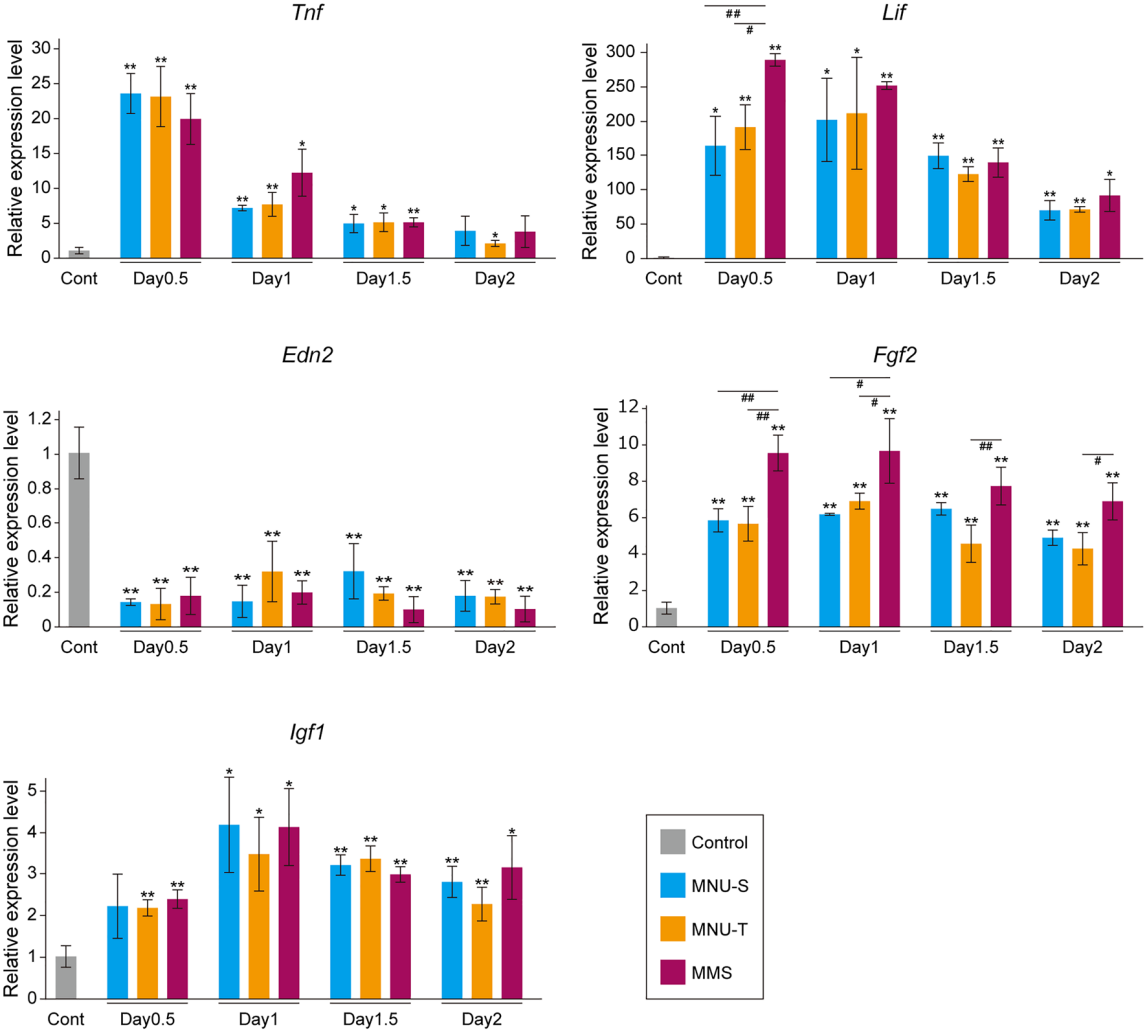
Gene expression of cytokines and growth factors after MNU- and MMS-induced retinal injury. qRT-PCR analyses of *Tnf*, *Lif*, *Edn2*, *Fgf2*, and *Igf1* after MNU and MMS treatments. Each bar represents the mean ± SD (n = 3) and the values expressed relative to controls (Cont) after normalization to *Gapdh* levels. *p < 0.05, **p < 0.01 (comparisons with control). ^#^*p* < 0.05, ^##^*p* < 0.01 (comparisons between treatments at the same stage).

It has been suggested that growth factors or cytokines produced by dying neurons or reactive Müller glia stimulate the proliferative and regenerative responses of Müller glia after retinal injury^12,13^. To gain further insight into the mechanism regulating the timing of Müller glia proliferation after injury, we next analyzed gene expression of five secretory factors (*Tnf*, *Lif*, *Edn2*, *Fgf2*, and *Igf1*), previously shown to promote Müller glia proliferation or reactivation^14–21^, by qRT-PCR. *Tnf* expression was significantly upregulated by day 0.5 after all treatments, but subsequently decreased thereafter (Fig. 4). The *Tnf* levels did not differ significantly between treatments at all stages examined (Fig. 4). *Lif* expression increased dramatically by day 0.5 after all treatments (Fig. 4). The *Lif* levels in the MMS-treated retinas were significantly higher than those in the MNU-treated retinas at day 0.5, but the differences were no more significant after day 1 (Fig. 4). *Edn2* expression reduced drastically by day 0.5 after all treatments and remained low during the period examined (Fig. 4). No statistically significant differences in the *Edn2* levels were observed between treatments at all stages examined (Fig. 4). *Fgf2* expression increased by day 0.5 after all treatments (Fig. 4). The *Fgf2* levels were significantly higher in the MMS-treated retinas compared the MNU-treated retinas at both day 0.5 and 1 (Fig. 4). The *Igf1* levels increased slightly after all treatments, but the changes were relatively small compared to the other factors and there were no statistically significant differences between treatments at all stages examined.

## Discussion

In the present study, we compared the effects of two alkylating agents, MNU and MMS, on the induction of photoreceptor degeneration and the subsequent injury responses in the rat retinas. We and others have previously reported that 70 mg/kg BW of MNU-S induces photoreceptor degeneration throughout the retina by day 2 after treatment^3^. However, the present results showed that 110 mg/kg BW of MNU-T was required to induce the same degree of photoreceptor degeneration. This indicates that the weight-based potency of MNU to induce retinal degeneration is varied between manufactures and caution has to be taken when using MNU from different sources. On the other hand, 75 mg/kg BW of MMS induced similar retinal degeneration to MNU-S, suggesting that the potency of MMS to induce retinal degeneration is comparable with MNU-S in weight-based comparison. Considering that MMS is less toxic (LD50 of acute toxicity by oral administration: 225 mg/kg (MMS) vs. 110 mg/kg (MNU))^22,23^ and easier to handle due to its high water-solubility and chemical stability, this agent may be an excellent alternative to MNU for chemical induction of photoreceptor degeneration.

Müller glia are the principal glial cells in the retina and have been reported to proliferate after retinal cell death^7^. We therefore examined the proliferative response of Müller glia after treatment with the alkylating agents. We first expected that cell cycle progression of Müller glia occurs in the same manner after all treatments as the timing and severity of photoreceptor degeneration appeared similar between treatments. However, expression of late G1/S phase regulators and BrdU incorporation revealed that Müller glia in the MMS-treated retinas entered S phase earlier than those in the MNU-treated retinas. Of note, the timing of G1 phase entry as shown by pRb phosphorylation was similar between treatments. This indicates the possibility that G1 phase length of Müller glia proliferation may vary depending on the alkylating agents used to induce photoreceptor degeneration. However, to determine the exact timing of cell cycle reentry is technically challenging and further investigations are required to precisely evaluate the G1 phase length of Müller glia proliferation. The different timings of S phase entry of Müller glia after different treatments prompted us to examine more precisely the temporal sequence of photoreceptor degeneration. The earlier occurrence of S phase cells after MMS treatment lead to the assumption that photoreceptor degeneration may initiate earlier after MMS treatment. However, contrary to this assumption, rod degeneration was observed at the same timing regardless of the treatment and cone degeneration was induced earlier by MNU than MMS. Given the previous reports that degenerating photoreceptors in zebrafish express TNFα, which promotes proliferation of Müller glia^14,15^, it is likely that the G1/S progression of Müller glia after MMS treatment may be facilitated by the presence of degenerating, but not dead, cone photoreceptors. Our findings suggest that cone photoreceptors may survive longer than rod photoreceptors after exposure to alkylating agents, consistent with the previous reports that cone photoreceptors have selective drug resistance to MNU-S^2,24^. The possibility that rods and cones may have different influences on cell cycle progression of Müller glia is a matter of great interest and warrants further investigation.

Various growth factors and cytokines are secreted in the retina after injury and are thought to regulate diverse injury responses, including neuroprotection, neurodegeneration, Müller glial proliferation and reactive gliosis^12,25,26^. We analyzed expression of five secretory factors in our photoreceptor injury models and observed drastic upregulation of *Tnf*, *Lif*, and *Fgf2*, slight upregulation of *Igf1*, and significant downregulation of *Edn2*. The downregulation of *Edn2* was unexpected given the previous reports indicating *Edn2* upregulation in degenerating photoreceptors^27,28^. It is possible that the induction of *Edn2* did not occur or occurred only transiently in our models, where photoreceptors die quickly by the effects of alkylating agents. *Tnf* and *Fgf2* have also been localized to degenerating photoreceptors^14,27,29,30^. However, these factors are also induced in Müller glia after injury^14,31,32^, and thus the observed upregulation of these factors may reflect the reactive response of Müller glia. Tnf, an inflammatory cytokine, has been reported to have both detrimental and protective effects on neurons in the retina^33–36^. Tnf has also been shown to activate Müller glial proliferation and neuronal regeneration in the zebrafish retina^14,15^. In mammals, however, although Tnf promotes proliferation and gliosis of Müller glia in vitro ^30,37,38^, its role in vivo in the regulation of Müller glia responses remains unexplored. *Tnf* expression was transiently upregulated after injury and its levels did not differ significantly between treatments at any time points examined. Thus, this factor is unlikely to contribute to the observed difference in the timing of Müller glia proliferation between the injury models. However, the cyclin D1 levels immediately after injury were similar between treatments, suggesting the possibility that Tnf induction may play a role in early G1 phase entry of Müller glia. In addition, Tnf is known to upregulate Lif and Fgf2^30,37–39^, and thus may indirectly regulate the Müller glial response via these downstream factors. Intriguingly, the expression of *Lif* and *Fgf2* was significantly higher in the MMS-treated retinas compared to the other treatments at day 0.5. Therefore, the *Lif* and *Fgf2* levels may be regulated by additional upstream factors other than *Tnf*. Both Lif and Fgf2 have been shown to increase in the injured retina and implicated in neuroprotection or reactive gliosis of Müller glia^16,29,40–42^. Mitogenic effects of Fgf2 on Müller glia have also been reported in zebrafish and chick^18–21^; however, its endogenous role in Müller glial proliferation in mammals remains unclear. The present results that *Lif* and *Fgf2* were more highly upregulated in the MMS-treated retinas, in which G1/S progression of Müller glia started earlier, strongly suggests the relevance of these factors in cell cycle progression of Müller glia. As the early (G0/G1 transition) and late stage (G1/S transition) of G1 phase may be regulated by distinct growth factors in a cell^43,44^, Lif and Fgf2 may act to drive the late G1 phase progression while factors, like Tnf, showing equivalent induction after all treatments may be involved in the activation of early G1 phase.

The present results revealed a similar time course of rod degeneration after all treatments. Considering that rods account for approximately 99% of all photoreceptors in the rat retina ^45,46^, it is puzzling that growth factor expression and Müller glia proliferation induced by photoreceptor injury varied between treatments. As cones seemed to survive longer after MMS treatment, it is tempting to speculate that cone-derived Fgf2 signaling may be responsible for the accelerated G1/S transition of Müller glia in the MMS-treated retinas. Alternatively, we cannot rule out the possibility that the higher levels of *Lif* and *Fgf2* may have delayed cone death in the MMS-treated retinas via their neuroprotective effects.

In conclusion, we compared the time course of photoreceptor injury, cell cycle progression of Müller glia, and expression of growth factors and cytokines in the rat retinas treated with different alkylating agents. Although causal associations between these events remain to be investigated, our data raise an interesting possibility that photoreceptor injury activates different regulatory factors linked to different phases of glial proliferation. The present photoreceptor degeneration models may be useful to reveal the complex regulatory mechanisms underlying the injury-induced proliferative response of Müller glia in vivo.

## Materials and methods

### Animals

Male Wistar rats (5 weeks old, 180–200 g of body weight) were obtained from the Charles River Laboratories Japan (Yokohama, Japan) and maintained under a 12:12 h light/dark cycle with free access to food and water. In all experiments, both eyes were enucleated at the same timing in daytime after euthanasia using CO_2_. All animal experiments were designed in accordance with the institutional code of ethics for laboratory animals and approved by the Institutional review board (Tokyo Women's Medical University, Approval No. AE23-043). The study was reported in accordance with ARRIVE guidelines.

### Induction of retinal degeneration

*N*-methyl-*N-*nitrosourea (MNU) was purchased from Sigma-Aldrich (St. Louis, MO, USA; referred as MNU-S) and Toronto Research Chemical (Toronto, Canada; referred as MNU-T) and stored at 4 °C and −20 °C, respectively. Methyl Methanesulfonate (MMS) was purchased from nacalai tesque (Kyoto, Japan) and stored at room temperature. These agents were dissolved directly in phosphate buffered saline (PBS) at 10 mg/ml (MNU) and 18.2 mg/ml (MMS) just before use. To induce photoreceptor degeneration in rats, 70 mg/kg BW of MNU-S, 70, 90, 110 mg/kg BW of MNU-T, and 45, 60, 75 mg/kg BW of MMS were administrated by intraperitoneal injection.

### Immunofluorescence

The eyes were fixed by 4% paraformaldehyde in PBS for 1 h, rinsed with 15% and 30% sucrose, and frozen in OCT compound (Leica Biosystems, Nussloch, Germany) with dry ice-isopentane. Serial 10 µm sections were prepared using a cryostat and subjected to immunofluorescence staining as previously reported^3^. Briefly, air-dried specimens were rinsed with 0.3% Triton-X100 in PBS (PBST), blocked with 10% normal donkey serum (Merck, Darmstadt, Germany) in PBST and incubated with primary antibodies, which were diluted with 10% bovine serum albumin (Sigma-Aldrich) in PBST, overnight at room temperature. Specimens were rinsed with PBST and incubated with diluted secondary antibodies for 30 min at room temperature, rinsed with PBST, and mounted with Fluoromount (Diagnostic BioSystem, Pleasanton, CA, USA). The primary and secondary antibodies used are listed in Supplementary Table S1. The cell nuclei were counterstained with 4', 6-diamidino-2phenylindole (DAPI; Sigma Aldrich). Immunofluorescence images were captured using a confocal laser scanning microscope LSM710 (Carl Zeiss, Jena, Germany).

### TUNEL assay

Apoptotic cells were labeled by terminal deoxynucleotidyl transferase-mediated dUTP nick end-labeling (TUNEL) with the in situ cell death detection kit, TMR red (Roche, Mannheim, Germany) following manufacturers instruction without any modifications.

### Cell counting

The vertically sliced retinas were imaged using a fluorescence microscope Eclipse E600 (Nikon Instruments, Tokyo, Japan) with a  20× objective lens and cells immunoreactive for specific cell markers were counted. Cell count was recorded for two fields of view per section excluding the peripheral portion of the retina, four sections per animal, and three animals per stage and condition (n = 3). Cell density was calculated per millimeter retina.

### Quantitative (real-time) RT-PCR

Total RNA was extracted from one retina collected from three animals per stage and condition (n = 3) using the RNeasy plus kit (QIAGEN, Hilden, Germany). After genomic DNA was removed using an RNase-free DNase set (QIAGEN), 100 ng of total RNA was reverse transcribed using the ReverTra Ace qPCR RT Master Mix (Toyobo, Osaka, Japan). Quantitative PCR was carried out by using PowerUp SYBR Green Master Mix (Thermo Fisher Scientific, Waltham, MA USA) in the Step One Plus real time PCR system (Thermo Fisher Scientific). Each reaction mix contained one hundre­dth of reverse transcription product (1 µl) and 0.4 µM of primers at the final concentration (10 µl reaction mix in total). Primers used are shown in Supplementary Table S2. Data were normalized to *Gapdh* expression and fold changes of each gene relative to control were represented by the mean of data from three independent samples.

### Statistical analysis

Statistical analysis of the difference between control and treatments was performed by Welch’s *t*-test by using the analysis tool on Excel (Microsoft, WA, USA). As for that between each treatment was performed by one-way analysis of variance (ANOVA) and Tukey–Kramer honestly significant difference (HSD) test using JMP software (SAS Institute, Cary, NC, USA). A value of *p* < 0.05 was considered statistically significant.

### Supplementary Information

Supplementary Information.

## Data Availability

The datasets generated and analyzed in this study are available from the corresponding author upon request.
