# Supplementary material for: Effects of different alkylating agents on photoreceptor degeneration and proliferative response of Müller glia
Source: Sci Rep. 2024 Jan 2;14:61. doi: 10.1038/s41598-023-50485-7 (PMC10762013; doi:10.1038/s41598-023-50485-7)
Supplement: Supplementary file 1 — Supplementary Information. [file 41598_2023_50485_MOESM1_ESM.pdf]

Manuscript Title:

Effects of different alkylating agents on photoreceptor degeneration and proliferative response of Müller glia.

Author names:

Kaori Nomura-Komoike, Reiko Nishino, and Hiroki Fujieda

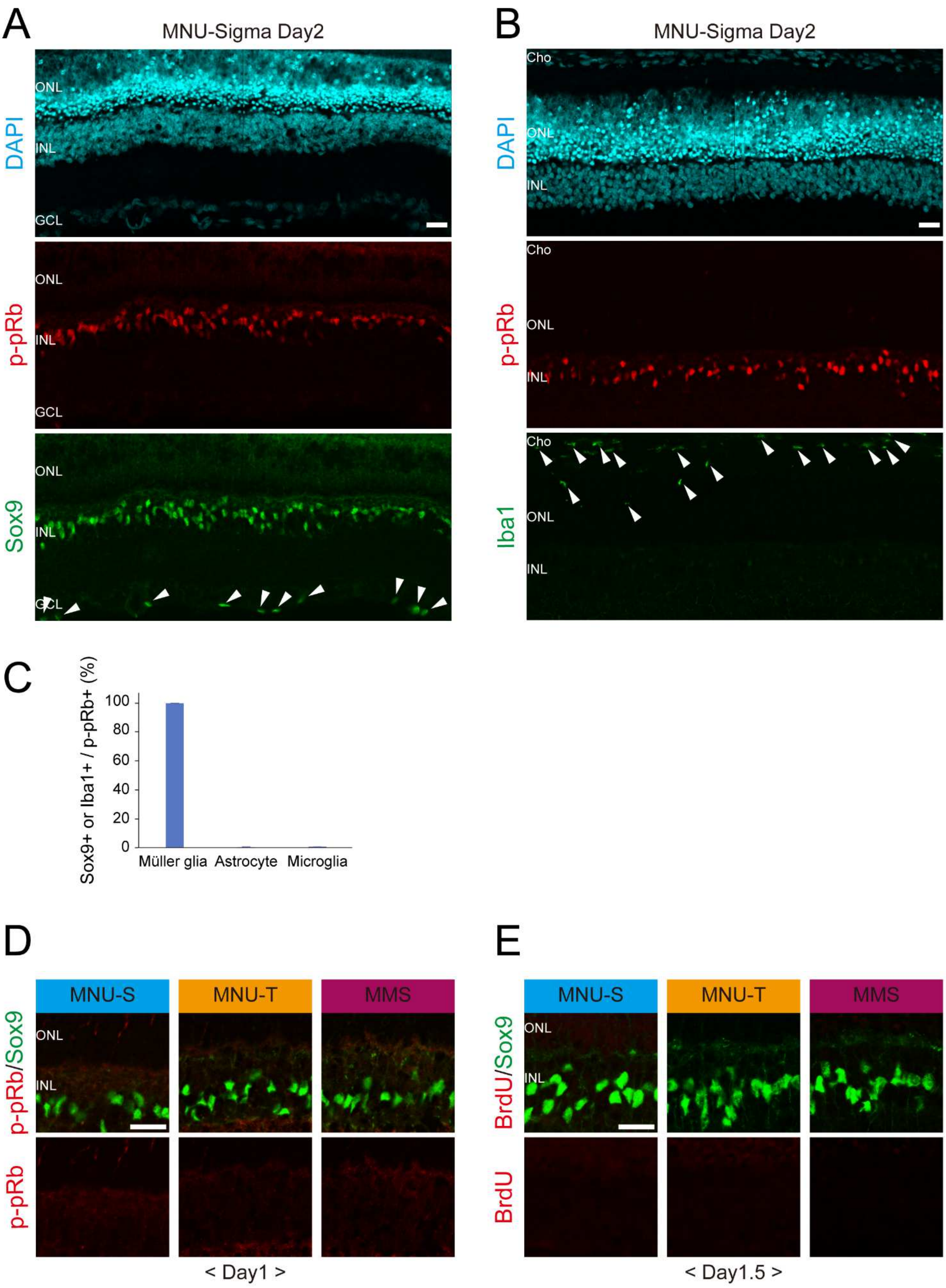

Supplementary Figure 1: Cell cycle reentry of Müller glia after injury as shown by phosphorylated retinoblastoma protein (p-pRb) immunofluorescence. (A) Double immunofluorescence for p-pRb (red) and Sox9 (green, Müller glia marker) at day 2 after MNU-S treatment. Note the absence of p-

pRb in Sox9+ astrocytes in the GCL (white arrowheads). (B) Double immunofluorescence for p-pRb (red) and Iba1 (green, microglia/macrophage marker) showing the absence of p-pRb in microglia/macrophages (white arrowheads). The nuclei were counterstained with DAPI (cyan). The horizontally-wide images shown in (A) and (B) were automatically taken by using the tile scan function in the confocal laser scanning microscope LSM710. (C) The proportions of p-pRb-positive Müller glia, astrocytes, and microglia/macrophages (n=3) calculated based on the staining shown in (A) and (B). (D) Double immunofluorescence for Sox9 (green) and p-pRb (red) at day 1 after treatments. (E) Double immunofluorescence for Sox9 (green) and bromodeoxyuridine (BrdU, red) at day 1.5 after treatments. Cho: choroid ONL: outer nuclear layer, INL: inner nuclear layer, GCL: ganglion cell layer. Scale bar= 20μm.

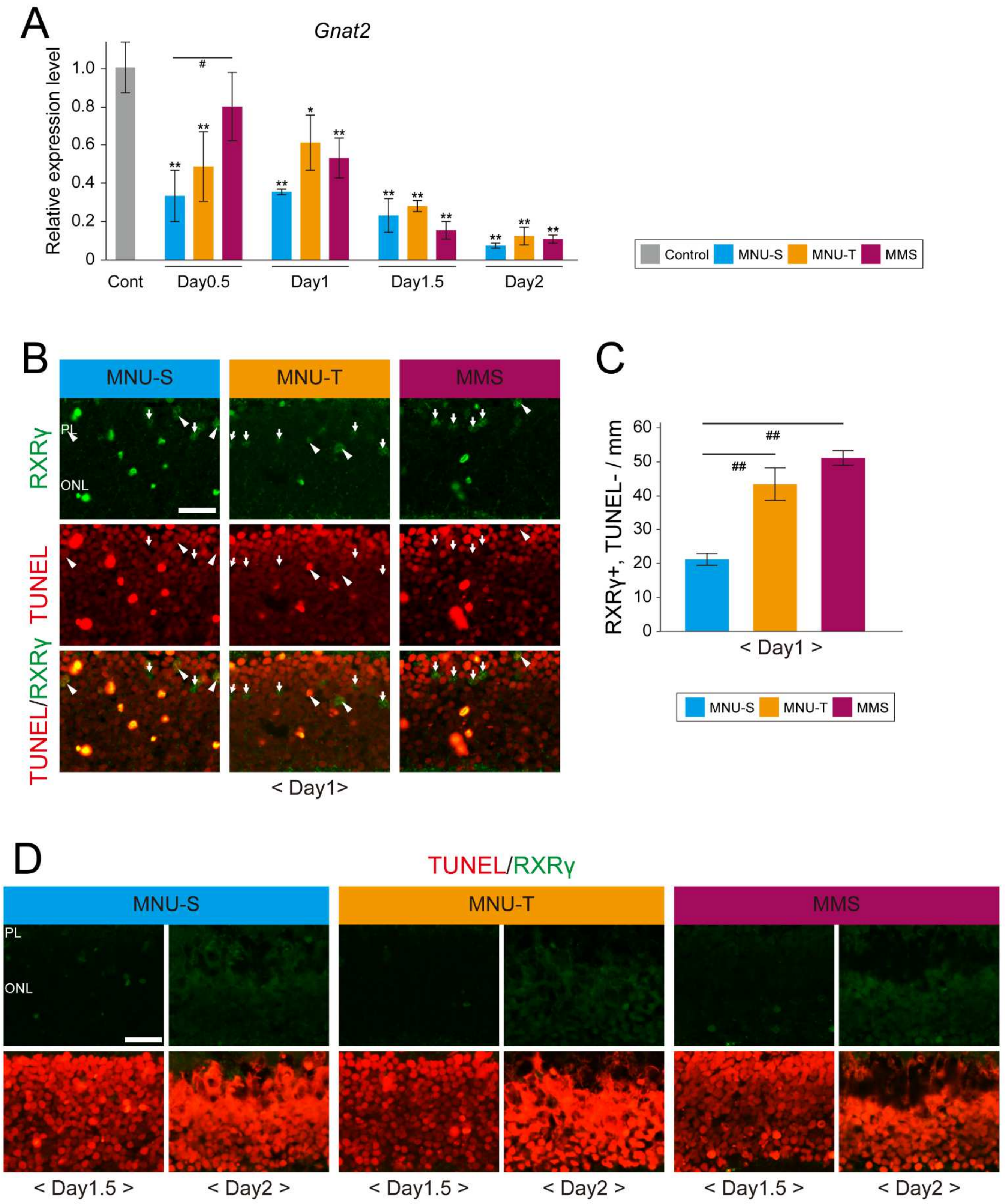

Supplementary Figure 2: (A) qRT-PCR analyses of *Gnat2* (cone-specific gene) after MNU and MMS treatments. Each bar represents the mean ± SD (n=3) and the values expressed relative to controls (Cont) after normalization to *Gapdh* levels. **p* < 0.05, ***p* < 0.01 (Comparisons with control), #*p* < 0.05 (Comparisons between treatments) (B) Immunofluorescence for RXRγ (green) in combination with TUNEL (red) at day 1 after treatment with alkylating agents. White arrowheads

indicate RXRγ/TUNEL double positive cells. White arrows indicate RXRγ single positive cells. (C) Quantification of surviving cone photoreceptors (RXRγ+, TUNEL-) at day 1 after MNU and MMS treatments. Each bar represents the mean ± SD (n=3). #$p < 0.05$, ##$p < 0.01$ (Comparisons between treatments). (D) Immunofluorescence for RXRγ (green) in combination with TUNEL (red) at day 1.5 and day 2, showing the absence of RXRγ signals. PL: photoreceptor layer, ONL: outer nuclear layer. Scale bar= 20µm.

Supplementary Table S1

Primary antibodies

Antibody	Dilution	Species	Source
Phospho-pRb	1000	Rabbit	Cell Signaling 9308
BrdU	500	Rat	Abcam ab6326
Sox9	2000	Rabbit	Millipore AB5535
Sox9	2000	Goat	R&D Systems AF3075
RXR γ	400	Rabbit	Santa cruz sc-555
Iba1	1000	Goat	Wako 011-27991

Secondary antibodies

Antibody	Dilution	Source
donkey anti-rabbit IgG Alexa Fluor 555	1000	Invitrogen A-31572
donkey anti-rabbit IgG Alexa Fluor 488	1000	Invitrogen A-21206
donkey anti-rat IgG Alexa Fluor 594	1000	Invitrogen A-21209
donkey anti-goat IgG Alexa Fluor 488	1000	Invitrogen A-11055

Supplementary Table S2

Primers

Gene	Forward	Reverse	Ref
Gapdh	ACAAGATGGTGAAGGTCGGTGTGA	AGCTTCCCATTCTCAGCCTTGACT	1
Ccnd1	CAGAAGTGCGAAGAGGAGGTC	TCATCTTAGAGGCCACGAACAT	1
Ccne1	GAAAAGCCAGGATAGCAGTCAG	CCCCAATTCAAGACGGGAAG	1
Ccne2	AGGAATCAGTCCTTGCATTATC	CCCAGCTTAAGTCTGGCAGAG	1
Ccna2	GCCTTCACCATTCATGTGGAT	TGGCTCCGGGTAAAGAGACAG	1
Rho	CCTACTTCCTGGCATGCTGAA	TCTGAGGCTCCAACCCATTC	1
Gnat1	CGGAAGCTGATGCACATGG	CTGAGGCTCGGTCAAAGCAA	1
Opn1sw	GTGAAGAAAAAGCAGCAGAC	TGGGCAGGGCAGCCATAGC	1
Opn1mw	TTGCTGACCTAGCAGAGACCA	AGCCTTCTATGACACACAGAG	1
Tnf	AAATGGGCTCCCTCTCATCAGTTC	TCTGCTTGGTGGTTTGCTACGAC	2
Lif	TCAACTGGCTCAACTCAACG	AAAGGTGGGAAATCCGTCAT	3
Edn2	GACTGCTGCGGGAGACCTT	GGGATGGCCTCTCTTGTCAA	4
Fgf2	AGTTGTGTCCATCAAGGGAGTGT	AGCCAGCAGCCGTCCAT	5
Igf1	TTCGGAGGGCACCACAGA	ACATCTCCAGCCTCCTCAGATC	6
Gnat2	CCACCTCAGTATCTGTTTCC	TCCTCATGTTGAGGTCAAGG	

References

1 Nomura-Komoike, K., Saitoh, F., Komoike, Y. & Fujieda, H. DNA Damage Response in Proliferating Müller Glia in the Mammalian Retina. *Invest Ophthalmol Vis Sci* **57**, 1169-1182 (2016). https://doi.org:10.1167/iovs.15-18101

2 Peinnequin, A. *et al.* Rat pro-inflammatory cytokine and cytokine related mRNA quantification by real-time polymerase chain reaction using SYBR green. *BMC Immunol* **5**, 3 (2004). https://doi.org:10.1186/1471-2172-5-3

3 Hu, Q., Huang, C., Wang, Y. & Wu, R. Expression of leukemia inhibitory factor in the rat retina following acute ocular hypertension. *Mol Med Rep* **12**, 6577-6583 (2015). https://doi.org:10.3892/mmr.2015.4287

4 Alrashdi, S. F., Deliyanti, D., Talia, D. M. & Wilkinson-Berka, J. L. Endothelin-2 Injures the Blood-Retinal Barrier and Macroglial Müller Cells: Interactions with Angiotensin II, Aldosterone, and NADPH Oxidase. *Am J Pathol* **188**, 805-817 (2018). https://doi.org:10.1016/j.ajpath.2017.11.009

5 Farmer, J. *et al.* Effects of voluntary exercise on synaptic plasticity and gene expression in the dentate gyrus of adult male Sprague-Dawley rats in vivo. *Neuroscience* **124**, 71-79 (2004). https://doi.org:10.1016/j.neuroscience.2003.09.029

6 Shingo, A. S. & Kito, S. Estrogen induces insulin-like growth factor-1 mRNA expression in the immortalized hippocampal cell: determination by quantitative real-time polymerase chain reaction. *Neurochem Res* **28**, 1379-1383 (2003). https://doi.org:10.1023/a:1024900616704
